# Clinical profiles and outcomes of different therapeutic protocols in elderly patients with trochanteric fractures: a descriptive study

**DOI:** 10.3389/fmed.2026.1789056

**Published:** 2026-02-24

**Authors:** Xiang Yu, Wei Wang, Feng Zhou, Xin-Yu Cao, Hai-Jian Lu, Hong-Kui Hu, Xu Li, Bing-Li Liu, Rong-Guang Ao

**Affiliations:** Department of Orthopedics, The Seventh People’s Hospital Affiliated to Shanghai University of Traditional Chinese Medicine, Shanghai, China

**Keywords:** conservative treatment, elderly patients, internal fixation, therapeutic protocols, trochanteric fractures

## Abstract

**Objective:**

To describe the clinical course and outcome profiles of elderly patients with trochanteric fractures, characterized by different baseline features, following different treatment pathways in clinical practice.

**Methods:**

This single-center retrospective descriptive study consecutively enrolled 309 elderly patients with trochanteric fractures admitted between January 2021 and December 2023. Based on the actual treatment received, patients were categorized into three groups: Group A (home-based recuperation, *n* = 81), Group B (inpatient conservative treatment, *n* = 97), and Group C (inpatient surgical treatment, *n* = 131). The baseline characteristics, treatment-related metrics, complication profiles, and functional recovery and survival status at one-year post-injury were collected and descriptively analyzed.

**Results:**

Treatment selection closely matched patients’ baseline health status. Patients in Group C were younger, had fewer comorbidities, and better baseline function; conversely, Group A patients were older, more frail, and had greater functional dependency. Group B patients’ characteristics were intermediate. Complication profiles differed among the groups: Group C was predominantly associated with surgery-related complications; Group B exhibited a combination of fracture healing issues and immobilization-related medical complications; Group A was most notably characterized by impaired fracture healing. The one-year survival rate observed among patients in Group C was 95.42%, which was associated with their more favorable baseline health status. Rates of 91.75% and 83.95% were observed in Group B and Group A, respectively, reflecting the gradient in baseline frailty across groups. Functional recovery scores showed a parallel distribution.

**Conclusion:**

This study delineates the distribution of outcomes following different treatment pathways in elderly intertrochanteric fracture patients with varying health statuses. It provides a crucial reference for individualized clinical decision-making and prognosis expectation management in this heterogeneous patient population.

## Introduction

With the accelerating global aging population, the incidence of hip fractures in the elderly continues to rise, posing a significant public health challenge. Among these, trochanteric fractures, as one of the most common types, are of particular concern due to their high mortality and disability rates (1). Currently, surgical intervention is the primary treatment for patients deemed medically fit, aiming to achieve early mobilization and functional recovery (2). However, a considerable number of patients in clinical practice are of advanced age, frail, and have multiple comorbidities, for whom the risks of surgery are significantly elevated, thus often leading to a shift toward conservative management (3). For this specific population, evidence regarding the most appropriate treatment strategy remains insufficient (4). Due to significant selection bias, traditional retrospective comparative studies struggle to accurately evaluate the true effects of different treatment modalities. Therefore, this study aims, through a descriptive design, to systematically present the clinical course and outcome profiles of elderly intertrochanteric fracture patients with different baseline characteristics who selected different treatment pathways (home-based recuperation, inpatient conservative treatment, inpatient surgical treatment). This aims to provide a reference for clinical decision-making, particularly for the individualized treatment of frail elderly patients.

## Materials and methods

### Study design

This is a retrospective descriptive study. Data were collected from 309 elderly patients with trochanteric fractures treated in our hospital’s orthopedic department between January 2021 and December 2023. Based on the treatment protocol, patients were divided into three groups: Group A (home-based recuperation), Group B (inpatient conservative treatment), and Group C (inpatient surgical treatment). Clinical characteristics and outcomes during treatment and up to one year post-injury were collected and compared across the groups. All patients provided informed consent, all procedures in this study adhered to the ethical principles of clinical research outlined in the Declaration of Helsinki, and the study was approved by the Medical Ethics Committee of our Hospital.

### Inclusion and exclusion criteria

Inclusion criteria: (1) Age ≥ 65 years. (2) A clear history of trauma. (3) Diagnosis of intertrochanteric fracture confirmed by X-ray or CT. Exclusion criteria: (1) Pathological fracture. (2) Old fracture. (3) Open fracture. (4) Patients with multiple injuries. (5) History of ipsilateral hip replacement surgery. (6) Combined major vascular or nerve injury. (7) Patients with incomplete follow-up data.

### Treatment protocols

- Group A (home-based recuperation): Patients and their families declined hospitalization and opted for conservative care at home or in a nursing institution. Emergency department physicians informed them of potential complications and management measures, prescribing non-steroidal anti-inflammatory drugs and anticoagulants.
- Group B (inpatient conservative treatment): Patients and their families agreed to hospitalization but refused surgery. Alternatively, patients with numerous underlying diseases were assessed by the medical team as unable to tolerate surgery. Patients in this group underwent skeletal traction via the tibial tuberosity on the affected limb, received low-molecular-weight heparin anticoagulation, and received active treatment for underlying conditions and prevention of various complications (5).
- Group C (inpatient surgical treatment): Patients were assessed by the medical team as suitable for surgery, and patients and their families consented to the procedure. Surgery involved closed reduction and internal fixation with Proximal Femoral Nail Antirotation (PFNA) system (Watson Medical Devices Co., Ltd., Zhejiang, China). The PFNA implant features a fixed cephalomedullary angle of 130°; the nail diameter and length were selected intraoperatively based on the patient’s femoral medullary cavity anatomy to achieve stable fixation. The goal of reduction was to achieve a stable configuration, defined as either anatomical reduction or, more commonly in osteoporotic fractures, positive medial cortex support (6), prioritizing mechanical stability over anatomical reduction to minimize surgical duration and blood loss in these elderly patients. Postoperative rehabilitation emphasized non-weight-bearing functional exercises initially, with progression based on individual tolerance and radiographic evidence of healing, aiming to prevent complications while avoiding excessive stress on the osteoporotic bone (5).

### Observation indicators

#### Baseline indicators

Collected patient information included: name, gender, age, fracture side, living situation (living alone/with family/in a nursing institution), and pre-fracture mobility status (independent walking/requiring walking aids/wheelchair-dependent/bedridden). Pre-fracture functional status was assessed using the Barthel Index for basic activities of daily living (BADL) (7) and the Lawton scale for instrumental activities of daily living (IADL) (8). The cause of injury was categorized as low-energy (e.g., ground-level fall) or high-energy (e.g., traffic accident). The treatment modality (home-based recuperation, inpatient conservative treatment, inpatient surgical treatment) was recorded. Additionally, bone mineral density (BMD), body mass index (BMI), Charlson Comorbidity Index (CCI) (9), and American Society of Anesthesiologists (ASA) physical status grade (10) were documented. Documented comorbidities (11, 12) included hypertension, coronary heart disease, chronic heart failure, history of stroke, diabetes, osteoporosis, dementia, Parkinson’s disease, chronic bronchitis, anemia, hypoproteinemia, chronic kidney disease, and malignancy. Fractures were classified according to the 2018 AO/OTA classification system (13).

#### Outcome indicators

Prognostic information included time from injury to surgery (days), operative duration (minutes), intraoperative blood loss (ml), transfusion volume (ml), length of hospital stay (days), short-term complications (7) (delirium, pulmonary infection, deep vein thrombosis, pulmonary embolism, urinary tract infection, pressure sore, surgical site infection, anemia, cardiovascular and cerebrovascular events), medium- to long-term complications (delayed union, non-union, malunion, implant loosening, implant fracture), discharge destination (home/nursing institution/rehabilitation hospital), fracture healing time (weeks), BADL at one year post-injury, IADL at one year post-injury, short physical performance battery (SPPB) at one year post-injury, timed up and go test (TUG) at one year post-injury. 1-year survival rate.

Patient basic information was registered at the first emergency department visit. Subsequently, telephone follow-ups were conducted at 2 weeks and 1 year post-injury to inquire about recovery from patients or their family members. As the study subjects were all elderly, with some having dementia, all selected indicators were objective and were scored based on observation by patients’ children or caregivers. Transfusion details, short-term complications, length of stay, and discharge destination were only recorded for Groups B and C. Time from injury to surgery, operative duration, intraoperative blood loss, implant loosening, and implant fracture were only recorded for Group C. Only comorbidities with a significant impact on surgery and fracture recovery were recorded. The formal assessment of short-term medical complications was limited to Groups B and C. This decision was made because for Group A (home-based recuperation), data collection would rely on non-professional caregivers, introducing unacceptable risks of recall and misclassification bias, thereby compromising data comparability and validity.

Patients were scheduled for outpatient follow-up visits starting at 2 months post-injury, recurring monthly (though not strictly fixed to specific dates), accompanied by family or caregivers. At each visit, X-rays were obtained to evaluate fracture healing. Radiographic union was defined as the presence of bridging callus, disappearance of the fracture line, and absence of pain or tenderness at the fracture site during weight-bearing or physical examination.

### Statistical analysis

All statistical analyses were performed using IBM SPSS Statistics (version 26.0, IBM Corp., Armonk, New York). Descriptive statistics were employed to summarize the characteristics of the study population. Continuous variables were assessed for normality using the Shapiro–Wilk test. Normally distributed data are presented as mean ± standard deviation (SD), while non-normally distributed data are expressed as median with interquartile range (IQR). Categorical variables are summarized as frequencies and percentages (*n*, %). For continuous variables, *P*-values were calculated using one-way analysis of variance (ANOVA). For categorical variables, *P*-values were calculated using the Chi-square test.

#### Statistical analysis rationale for a descriptive study

As this is a descriptive study aimed at characterizing patient profiles and outcome distributions rather than testing causal hypotheses, all statistical analyses were strictly descriptive. The use of inferential statistics (e.g., ANOVA for continuous variables, chi-square tests for categorical variables) was solely to quantify the magnitude of baseline differences between groups and to confirm the expected heterogeneity in patient selection. No formal comparative analyses of outcomes were performed, as any observed differences in outcomes would be confounded by systematic baseline disparities. All *P*-values reported in this study should be interpreted as indicators of baseline imbalance, not as evidence of treatment effects.

## Results

### Baseline characteristics

**FIGURE 1:**
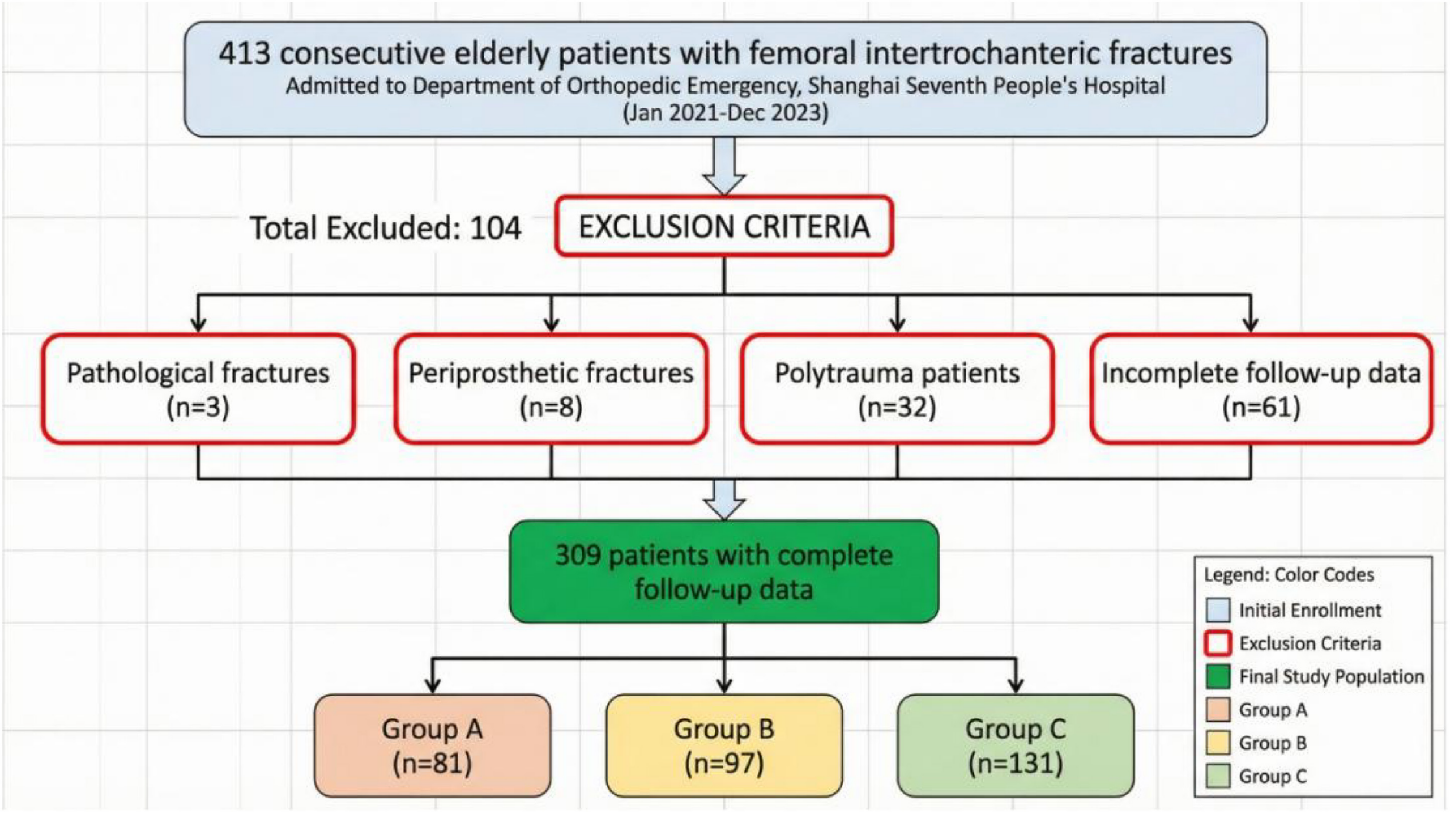
Flowchart.

**FIGURE 2:**
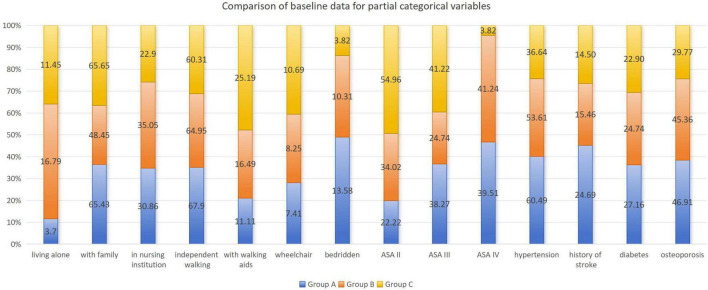
Comparison of baseline data for partial categorical variables.

**FIGURE 3:**
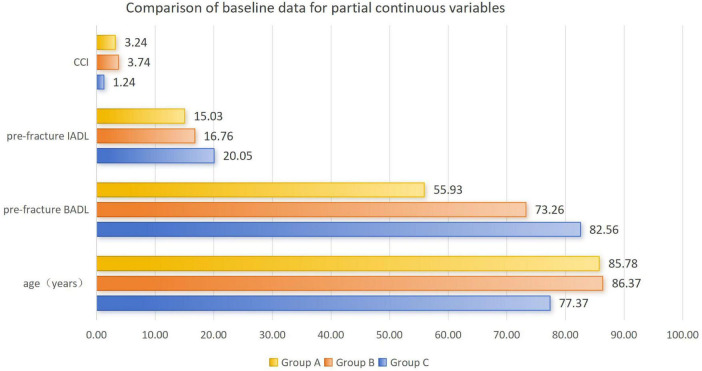
Comparison of baseline data for partial continuous variables.

A total of 309 elderly patients with trochanteric fractures were ultimately included in this study, with the screening illustrated in Figure 1. All patients were divided into Group A (home-based recuperation, *n* = 81), Group B (inpatient conservative treatment, *n* = 97), and Group C (inpatient surgical treatment, *n* = 131) based on treatment pathway. Detailed comparisons of baseline characteristics are presented in Figures 2, 3 and Table 1 .

**TABLE 1:** Baseline characteristics.

Included	Total, *N* = 309	Group A, *N* = 81	Group B, *N* = 97	Group C, *N* = 131	*P*-value
Gender					0.74
Male, *N* (%)	104 (33.66)	30 (37.04)	31 (31.96)	43 (32.82)	
Female, *N* (%)	205 (66.34)	51 (62.96)	66 (68.04)	88 (67.18)	
Age (years), mean ± SD	82.56 ± 10.47	85.78 ± 8.89	86.37 ± 9.34	77.37 ± 7.74	0.00*
Fracture side					0.29
Left, *N* (%)	148 (47.90)	42 (51.85)	50 (51.55)	56 (42.75)	
Right, *N* (%)	161 (52.10)	39 (48.15)	47 (48.45)	75 (57.25)	
Living situation					0.01*
Living alone, *N* (%)	34 (11.00)	3 (3.70)	16 (16.79)	15 (11.45)	
With family, *N* (%)	186 (60.19)	53 (65.43)	47 (48.45)	86 (65.65)	
In nursing institution, *N* (%)	89 (28.80)	25 (30.86)	34 (35.05)	30 (22.90)	
Pre-fracture mobility					0.04*
Independent walking, *N* (%)	197 (63.75)	55 (67.90)	63 (64.95)	79 (60.31)	
With walking aids, *N* (%)	58 (18.77)	9 (11.11)	16 (16.49)	33 (25.19)	
Wheelchair, *N* (%)	28 (9.06)	6 (7.41)	8 (8.25)	14 (10.69)	
Bedridden, *N* (%)	26 (8.41)	11 (13.58)	10 (10.31)	5 (3.82)	
Pre-fracture BADL, Mean ± SD	72.45 ± 21.88	55.93 ± 16.91	73.26 ± 17.37	82.56 ± 11.66	0.00*
Pre-fracture IADL, mean ± SD	16.26 ± 7.21	15.03 ± 7.22	16.76 ± 5.67	20.05 ± 4.79	0.00*
Cause of injury					0.27
Low-energy, *N* (%)	295 (95.47)	79 (97.53)	90 (92.78)	126 (96.18)	
High-energy, *N* (%)	14 (4.53)	2 (2.47)	7 (7.22)	5 (3.82)	
BMD (T), mean ± SD	-1.86 ± 0.85	-1.75 ± 0.78	-1.68 ± 0.99	-1.79 ± 0.81	0.20
BMI (kg/m^2^), Mean ± SD	22.81 ± 1.92	23.11 ± 1.78	22.88 ± 2.04	21.61 ± 2.22	0.32
CCI, mean ± SD	3.84 ± 1.53	3.24 ± 1.91	3.74 ± 1.71	1.24 ± 0.83	0.00*
ASA grade					0.00*
I, *N* (%)	0 (0)	0 (0)	0 (0)	0 (0)	
I级, *N* (%)	123 (39.81)	18 (22.22)	33 (34.02)	72 (54.96)	
III, *N* (%)	109 (35.28)	31 (38.27)	24 (24.74)	54 (41.22)	
IV, *N* (%)	77 (24.92)	32 (39.51)	40 (41.24)	5 (3.82)	
V, *N* (%)	0 (0)	0 (0)	0 (0)	0 (0)	
**Comorbidities**					
Hypertension, *N* (%)	149 (48.22)	49 (60.49)	52 (53.61)	48 (36.64)	0.00*
Coronary heart disease, *N* (%)	44 (14.24)	14 (17.28)	14 (14.43)	16 (12.21)	0.59
Chronic heart failure, *N* (%)	48 (15.53)	14 (17.28)	15 (15.46)	19 (14.50)	0.86
History of stroke, *N* (%)	54 (17.48)	20 (24.69)	15 (15.46)	19 (14.50)	0.00*
Diabetes, *N* (%)	76 (24.60)	22 (27.16)	24 (24.74)	30 (22.90)	0.01*
Osteoporosis, *N* (%)	121 (39.16)	38 (46.91)	44 (45.36)	39 (29.77)	0.01*
Dementia, *N* (%)	16 (5.18)	6 (7.41)	7 (7.22)	3 (2.29)	0.14
Parkinson’s disease, *N* (%)	9 (2.91)	4 (4.94)	3 (3.09)	2 (1.53)	0.35
Chronic bronchitis, *N* (%)	99 (32.04)	29 (35.80)	28 (28.87)	42 (32.06)	0.61
Anemia, *N* (%)	58 (18.77)	16 (19.75)	16 (16.49)	26 (19.85)	0.79
Hypoproteinemia, *N* (%)	38 (12.30)	16 (19.75)	9 (9.28)	13 (9.92)	0.13
Chronic kidney disease, *N* (%)	26 (8.41)	7 (8.64)	9 (9.28)	10 (7.63)	0.90
Malignancy, *N* (%)	15 (4.85)	5 (6.17)	5 (5.15)	5 (3.82)	0.73
AO/OTA classification					0.35
A1, *N* (%)	208 (67.31)	60 (74.07)	62 (63.92)	86 (65.65)	
A2, *N* (%)	67 (21.68)	11 (13.58)	25 (25.77)	31 (23.66)	
A3, *N* (%)	34 (11.00)	10 (12.35)	10 (10.31)	14 (10.69)	

BADL, basic activities of daily living; IADL, instrumental activities of daily living; BMD, bone mineral density; BMI, body mass index; CCI, Charlson Comorbidity Index; ASA, American Society of Anesthesiologists,*Statistically significant difference at *P* < 0.05.

- Demographic and basic health characteristics: The mean age of the entire cohort was 82.56 ± 10.47 years, with a female predominance (66.34%). The mean age of Group C patients (77.37 ± 7.74 years) was significantly lower than that of Group A (85.78 ± 8.89 years) and Group B (86.37 ± 9.34 years) (*P* < 0.001). The CCI score was significantly lower in Group C (1.24 ± 0.83) compared to Group A (3.24 ± 1.91) and Group B (3.74 ± 1.71) (*P* < 0.001). The distribution of ASA grades differed significantly (*P* < 0.001), with ASA Grade II being most prevalent in Group C (54.96%), while Grades III and IV were more common in Groups A and B. No significant differences were observed among the groups regarding gender, fracture side, bone mineral density, or BMI (*P* > 0.05).
- Living status and functional level: Significant differences existed among the three groups in living arrangements (*P* = 0.01), although cohabitation with family was the predominant mode (60.19%) overall. Pre-fracture mobility showed a statistically significant difference among groups (*P* = 0.04). Both BADL (82.56 ± 11.66) and IADL (20.05 ± 4.79) scores were significantly higher in Group C compared to Group A (BADL: 55.93 ± 16.91; IADL: 15.03 ± 7.22) and Group B (BADL: 73.26 ± 17.37; IADL: 16.76 ± 5.67) (*P* < 0.001). The distribution of injury mechanism did not differ significantly among groups (*P* = 0.27).
- Comorbidities and fracture classification: The prevalence of hypertension (*P* < 0.001), history of stroke (*P* < 0.001), diabetes (*P* = 0.01), and osteoporosis (*P* = 0.01) differed significantly among the three groups, with higher rates in Groups A and B compared to Group C. However, no statistically significant differences were found for other comorbidities such as coronary heart disease, chronic heart failure, dementia, Parkinson’s disease, etc (*P* > 0.05). The distribution of AO/OTA fracture classification did not differ significantly among the groups (*P* = 0.35).

### Outcome measurements

**FIGURE 4:**
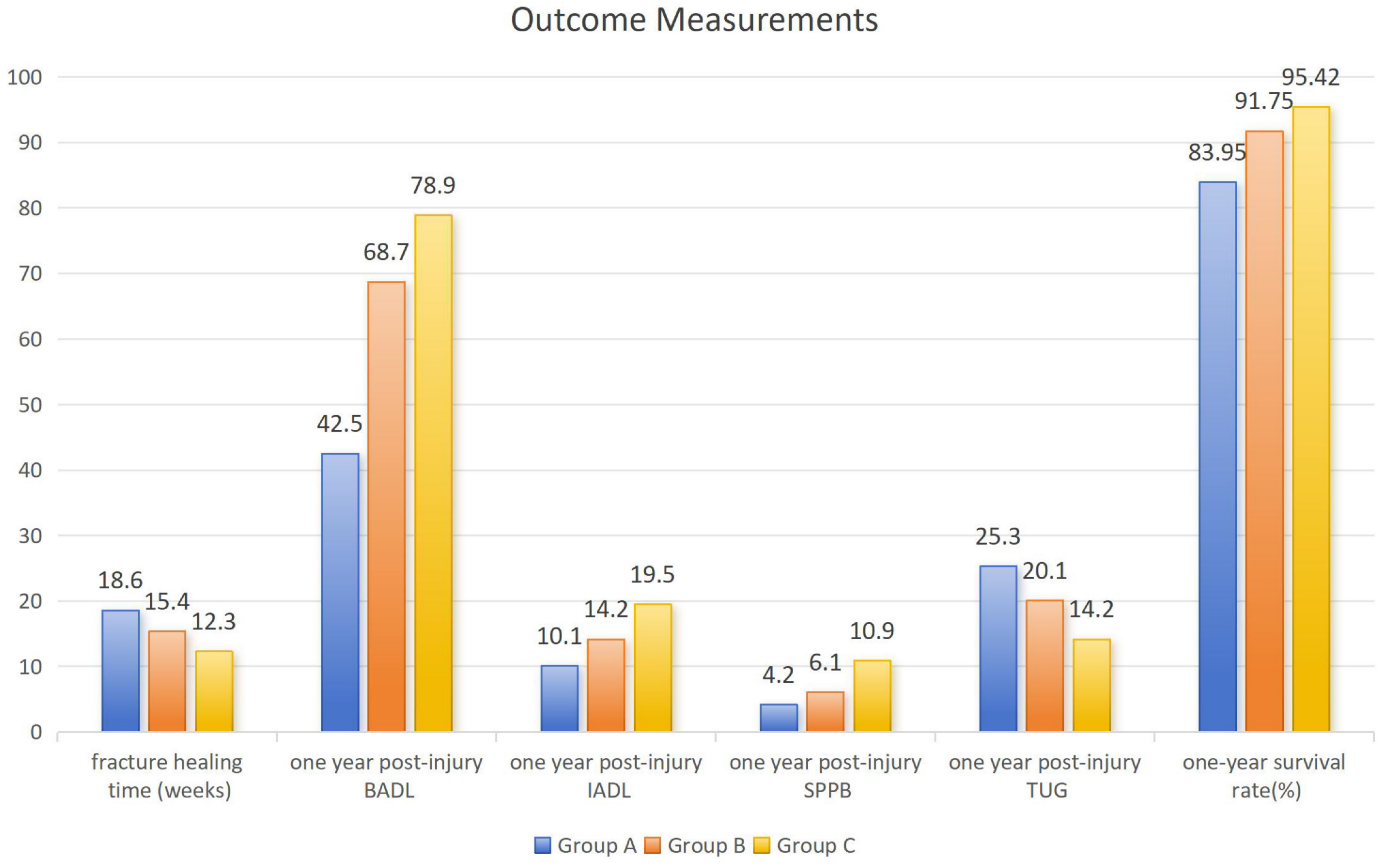
Outcome measurements.

Outcome data for the 309 patients and each subgroup are presented in Figure 4 and Table 2. The mean fracture healing time was 14.2 ± 4.8 weeks, and the 1-year survival rate was 91.26%. Common short-term complications included delirium (11.33%), anemia (16.18%), and urinary tract infection (6.47%). Among medium- to long-term complications, the rates were 13.59% for delayed union, 9.06% for non-union, and 24.92% for malunion. The 1-year follow-up showed mean BADL and IADL scores for the entire cohort of 65.2 ± 18.4 and 15.8 ± 8.1, respectively. For functional assessments, the mean SPPB score was 7.4 ± 2.9, and the mean TUG test result was 18.6 ± 8.4 s. Discharge destination analysis indicated that 79.29% of patients returned home or to a nursing institution, while 20.71% were transferred to a rehabilitation hospital.

**TABLE 2:** Outcome measures.

Included, *N* (%)	Total, *N* = 309	Group A, *N* = 81	Group B, *N* = 97	Group C, *N* = 131
Time from injury to surgery (days), mean ± SD	—	—	—	3.14 ± 1.56
Operative duration (minutes), mean ± SD	—	—	—	45.56 ± 10.66
Intraoperative blood loss (ml), mean ± SD	—	—	—	56.22 ± 15.09
Transfusion volume (ml), mean ± SD	—	—	200 ± 120	320 ± 180
Length of hospital stay (days), mean ± SD	—	—	18.4 ± 10.6	16.7 ± 4.9
**Short-term complications**				
Delirium, *N* (%)	—	—	12 (12.37)	23 (17.55)
Pulmonary infection, *N* (%)	—	—	12 (12.37)	6 (4.58)
Deep vein thrombosis, *N* (%)	—	—	8 (8.25)	8 (6.11)
Pulmonary embolism, *N* (%)	—	—	2 (2.06)	4 (3.05)
Urinary tract infection, *N* (%)	—	—	13 (13.40)	7 (5.34)
Pressure sore, *N* (%)	—	—	6 (6.19)	4 (3.05)
Surgical site infection, *N* (%)	—	—	—	7 (5.34)
Anemia, *N* (%)	—	—	18 (18.56)	32 (24.43)
Cardiovascular and cerebrovascular events, *N* (%)	—	—	5 (5.15%)	11 (8.40)
**Medium- to long-term complications**				
Delayed union, *N* (%)	42 (13.59)	25 (30.86)	12 (12.37)	5 (3.82)
Non-union, *N* (%)	28 (9.06)	18 (22.22)	7 (7.22)	3 (2.29)
Malunion, *N* (%)	77 (24.92)	64 (79.01)	9 (9.28)	4 (3.05)
Implant loosening, *N* (%)	—	—	—	4 (3.05)
Implant fracture, *N* (%)	—	—	—	1 (0.76)
**Discharge destination**				
Home/nursing institution, *N* (%)	245 (79.29)	72 (88.89)	65 (67.01)	108 (82.44)
Rehabilitation hospital, *N* (%)	64 (20.71)	9 (11.11)	32 (32.99)	23 (17.56)
Fracture healing time (weeks), Mean ± SD	14.2 ± 4.8	18.6 ± 5.2	15.4 ± 4.1	12.3 ± 3.2
BADL at one year post-injury	65.2 ± 18.4	42.5 ± 20.1	68.7 ± 15.3	78.9 ± 12.6
IADL at one year post-injury	15.8 ± 8.1	10.1 ± 7.8	14.2 ± 6.9	19.5 ± 5.4
SPPB at one year post-injury	7.4 ± 2.9	4.2 ± 2.4	6.1 ± 2.1	10.9 ± 2.0
TUG at one year post-injury	18.6 ± 8.4	25.3 ± 10.2	20.1 ± 7.8	14.2 ± 5.7
One-year survival rate, *N* (%)	282 (91.26)	68 (83.95)	89 (91.75)	125 (95.42)

BADL, basic activities of daily living; IADL, instrumental activities of daily living; SPPB, short physical performance battery; TUG, timed up and go test.

## Discussion

This study aimed to systematically describe treatment pathways and outcomes in elderly intertrochanteric fracture patients, based on clinical decisions. Unlike analytical studies designed to test hypotheses, this study focused on comprehensive observation and documentation of current clinical practice, rather than comparing the superiority of different treatments or making causal inferences. As treatment group allocation was not randomized but resulted from non-random, real-world decisions based on patients’ baseline health status, personal preferences, and clinical assessment, there were systematic and clinically meaningful differences in key baseline indicators such as age, comorbidities, and functional status among the groups (Table 1). This inherent selection bias means that any differences in outcomes cannot be simply attributed to the treatment modality itself. Therefore, this study deliberately avoided formal statistical comparisons of outcome measures (Table 2). All presented results should be regarded as descriptive reports of typical prognostic features under different clinical pathways. Its scientific value lies in providing a real-world spectrum of outcomes for the clinical management of this special population and establishing a baseline for subsequent targeted research.

The methodological design of this study was systematically optimized for the particularities of elderly hip fracture patients, primarily reflected in three key aspects: first, in the selection of assessment indicators, we moved away from the Harris Hip Score, which requires professional medical personnel, and instead adopted BADL and IADL as primary functional evaluation tools (14). This innovation allowed patients’ daily caregivers to participate in the assessment process, significantly enhancing the feasibility of long-term follow-up in community and home settings. Second, in terms of data collection methods, we fully considered the mobility challenges of elderly hip fracture patients and employed telephone follow-up as the primary method. This strategy effectively reduced the loss to follow-up rate due to geographical barriers and physical limitations, ensuring data completeness (15). The reliability of telephone follow-up is further supported by its cost-effectiveness and ability to identify psychosocial needs in elderly populations, as evidenced in previous studies (16). Finally, regarding indicator attributes, we strictly selected objective clinical indicators (e.g., radiological evidence of union, survival rate, readmission rate), minimizing measurement errors associated with cognitive impairment in elderly patients (17). These targeted methodological improvements collectively form a research framework suitable for the frail elderly population, providing methodological support for obtaining high-quality clinical research evidence.

The study results showed a high degree of concordance between the chosen treatment pathway and the patients’ baseline health status. Specifically, patients with the best baseline status (Group C, mean age 77.4 years, CCI 1.2, preoperative BADL 82.6) predominantly received surgical treatment (18, 19); whereas patients with the poorest baseline status (Group A, mean age 85.8 years, CCI 3.2, preoperative BADL 55.9) more often opted for home-based recuperation or conservative treatment. This finding is consistent with a Norwegian study on hip fracture pathways, which reported that younger and healthier patients were more likely to be assigned to home-based or specialized rehabilitation pathways, while older and frailer patients tended to receive conventional rehabilitation or nursing home care, highlighting the universal practice of risk-stratified decision-making (20). This “tailor-made” clinical decision-making pattern fully embodies the core principle of individualized treatment for elderly hip fractures—namely, comprehensive assessment based on the patient’s physiological reserve, functional status, and surgical risk, rather than deciding solely based on fracture type (no significant difference in AO/OTA classification distribution among the three groups). This risk-stratified decision logic, on one hand, reflects respect for the heterogeneity of elderly patients in clinical practice, and on the other hand, explains that the observed outcome differences among the treatment groups (e.g., lowest complication rate and best functional recovery in Group C) are largely attributable to patient selection bias rather than the absolute superiority of the treatment modality itself.

The pattern of outcome differences revealed in this study provides important implications for clinical decision-making. Although causal inference is limited by the descriptive design, the regular outcome differences observed across the three groups clearly delineate three distinct clinical pathways and their general outcomes: for patients whose physical condition permits (Group C), surgical treatment is an active and effective pathway (21). Patients in this group, who were selected for surgery based on their better baseline status, were observed to have? the shortest fracture healing time (12.3 weeks), a 1-year survival rate of 95.42%, and the highest functional recovery scores (BADL: 78.9). These outcomes are consistent with the expected prognosis for a healthier patient population. For high-risk patients unable to undergo surgery but receiving medical supervision (Group B), inpatient conservative treatment provided vital life support (22). Although functional recovery in this group was intermediate, their 1-year survival rate (91.75%) was notably higher than the rate observed in Group A (83.95%). This difference underscores the potential impact of inpatient medical supervision even when surgery is not feasible, within the context of differential baseline risks. This indicates that even when surgery is not feasible, active inpatient medical intervention (e.g., managing complications, professional nursing care) is crucial for stabilizing the condition and prolonging survival. This finding is supported by a multicenter cohort study which demonstrated that non-operative management for frail elderly patients with limited life expectancy yielded non-inferior quality-of-life outcomes (EQ-5D utility scores) compared to surgical management, with lower rates of adverse events, highlighting its viability as an alternative to surgery in this population (3). The outcomes observed in Group A, which comprised the frailest patients who forewent inpatient treatment, were the least favorable. This highlights that disengagement from systematic medical supervision may carry significantly higher risks for this particularly vulnerable population (23). The trend of all outcome indicators in this group was the least favorable, particularly the 1-year survival rate (83.95%) and functional recovery levels were considerably lower than the other two groups. This highlights that for these patients, even without surgery, disengagement from systematic medical supervision may carry significantly higher risks (24). These findings provide valuable prognostic references for treatment pathway selection in elderly patients with different health statuses.

The differences in complication profiles observed in this study clearly outline the distinct risk contours specific to each treatment pathway. Complications in Group C were primarily concentrated in surgery-related areas, such as local issues like implant loosening, failure, and surgical site infection, reflecting the unavoidable technical risks of invasive procedures (25); however, due to early mobilization post-surgery, the incidence of systemic medical complications (e.g., hypostatic pneumonia, deep vein thrombosis) was relatively low (26). Group B presented a dual-high-risk situation: on one hand, the lack of stable fracture fixation led to a significant increase in orthopedic complications like malunion and non-union; on the other hand, prolonged immobilization induced a series of medical complications such as pulmonary infection, urinary tract infection, and pressure sores (27). Group A, having received no definitive treatment, was most prominently characterized by fracture healing impairment, with rates of delayed union, non-union, and malunion significantly higher than the other groups. Concurrently, the timely detection and management of various complications faced challenges due to the lack of professional medical monitoring (28). This differentiated pattern of complication distribution indicates that clinical decision-making essentially involves weighing different risk spectra: surgery primarily bears technique-related risks, conservative treatment faces the dual pressure of orthopedic and medical complications, while home-based recuperation bears the compound risk of poor fracture healing and insufficient complication management. Identifying and understanding these risk spectra are of great importance for guiding individualized treatment decisions.

While the absolute outcome rates (e.g., specific survival percentages or complication frequencies) may vary across institutions due to differences in patient demographics and healthcare resources, the fundamental pattern of risk-stratified decision-making observed in our study is likely to have broad external validity. The consistent alignment between treatment selection and baseline frailty status reflects a universal clinical approach to managing heterogeneous elderly populations. This suggests that the key finding—that treatment pathways are tailored to individual patient characteristics rather than being applied uniformly—may be generalizable across diverse settings, even if the specific outcomes differ.

### Limitations

This study is a single-center retrospective descriptive study. Its core limitation lies in the significant differences in baseline characteristics (e.g., age, comorbidities, baseline function) among groups due to the non-randomized design; therefore, the observed outcome differences primarily reflect patient population heterogeneity rather than the causal effects of the treatment modalities. Secondly, some outcome indicators are group-specific (e.g., implant complications apply only to the surgical group); while scientifically reasonable, this limits inter-group comparability. Furthermore, functional assessments conducted via telephone follow-up and answered by family members may be subject to recall bias. Finally, single-center data may also affect the generalizability of the conclusions. These limitations must be fully considered when interpreting the descriptive results.

## Conclusion

This descriptive study systematically presents new treatment pathways and associated outcome spectra of elderly patients with trochanteric fractures of varying health statuses in clinical practice. The study found that treatment selection was highly matched with patients’ baseline health status: patients with better functional status and fewer comorbidities mostly underwent surgery and had the best prognosis; older, frailer patients with more comorbidities tended toward conservative treatment or home-based recuperation, with correspondingly higher prognostic risks. The three treatment pathways exhibited characteristic complication profiles: the surgery group was dominated by internal fixation-related issues, the conservative treatment group combined fracture healing problems with risks of immobilization-related complications, and the home-based recuperation group was most notable for fracture healing impairment. The outcome spectrum provided by this study offers an important reference for clinical expectation management, emphasizing the importance of tailoring treatment decisions based on individual patient circumstances.

## Data Availability

The raw data supporting the conclusions of this article will be made available by the authors, without undue reservation.
